# Feasibility of transferring intensive cared preterm infants from incubator to open crib at 1600 grams

**DOI:** 10.1186/1824-7288-40-41

**Published:** 2014-05-03

**Authors:** Giovanni Barone, Mirta Corsello, Patrizia Papacci, Francesca Priolo, Costantino Romagnoli, Enrico Zecca

**Affiliations:** 1 Division of Neonatology, Department of Pediatrics, Catholic University of the Sacred Heart, Rome, Italy

**Keywords:** Thermoregulation, Weaning, Discharge, Length of stay

## Abstract

**Background:**

Ability to maintain a normal body temperature in an open crib is an important physiologic competency generally requested to discharge preterm infants from the hospital. The aim of this study is to assess the feasibility of an early weaning protocol from incubator in preterm newborns in a Neonatal Intensive Care Unit.

**Methods:**

101 infants with birth weight < 1600 g were included in this feasibility study. We compared 80 newborns successfully transferred from an incubator to open crib at 1600 g with 21 infants transferred at weight ≥ 1700 g. The primary outcome was to evaluate feasibility of the protocol and the reasons for the eventual delay. Secondary outcomes were the identification of factors that would increase the likelihood of early weaning, the impact of an earlier weaning on discharge timing, and the incidence of adverse outcomes. Newborns in the study period were then compared with an historical control group with similar characteristics.

**Results:**

Early weaning was achieved in 79.2% of infants without significant adverse effects on temperature stability or weight gain. Delayed weaning was mainly due to the need of respiratory support. Gestational age affected the likelihood of early weaning (OR 1.7282 95% CI: 1.3071 - 2.2850). In the multivariate linear regression, early weaning reduced length of stay (LOS) by 25.8 days (p < 0.0001).

**Conclusions:**

Preterm infants can be weaned successfully from an incubator to an open crib at weight as low as 1600 grams without significant adverse effect. Early weaning significantly reduces LOS in preterm newborns.

## Background

Abilities to maintain a normal body temperature in an open crib, to suckle feed, to maintain stable cardio-respiratory function and to grow at an acceptable rate, are the physiologic competencies generally requested to discharge preterm infants from the hospital. The majority of preterm infants achieves these competencies between 34 and 36 weeks postmenstrual age [1]. A target weight of 1800–2000 grams is often chosen for incubator weaning, but this cut-off is largely based on the professional experience of clinicians and varies widely among neonatal units [2]. Assistance in open crib can induce a more positive maternal attitude, and promotes parental bonding and breastfeeding [3]. On the other hand, delaying transition to an open crib may results in hospitalization longer than necessary, consequently increasing the family discomfort and the cost of care. A randomised clinical trial, conducted in the neonatal sub-intensive ward of our university hospital, demonstrated that an early weaning protocol from incubator at a body weight of 1600 g versus 1800 g was safe and reduced the median length of stay (LOS) of 9.5 days in moderately preterm infants [4]. New et al. recently reported that medically stable preterm infants could be transferred to open cribs at a weight of 1600 g without significant adverse effects on temperature stability or weight gain [5]. However, in this study 814 newborns were assessed for eligibility but only 183 infants were actually randomized, so this results should be interpreted with caution and could not be safely extended to a wider population of preterm infants. The aims of our study were to verify the feasibility of an early weaning protocol from incubator in all preterm infants admitted to NICU, to analyze reasons for the eventual delayed transfer, and to evaluate the impact of an earlier transfer to open crib on discharge timing.

## Methods

### Design and setting

This non-randomized feasibility study was conducted between July 1, 2009 and December 31, 2010 in the NICU of our university hospital with the approval of the Ethical Committee of our Department of Pediatrics. Written consent was obtained from parents. Infants with major congenital abnormalities at birth were excluded from the study, while all other infants with birth weight (BW) < 1600 g were eligible for being transferred from incubator to open crib as soon as they reached 1600 g. No further assessment was required by the assisting team, before deciding to start the weaning protocol. Weaning was defined as early (early weaning EW) if an infant was transferred to open crib when his/her weight was between 1.600 and 1699 g, or delayed (delayed weaning DW) if transition was delayed at weight ≥ 1700 g.

### Outcomes and sample size

The primary outcome was to evaluate feasibility of EW in all eligible infants, and to analyze reasons for the eventual DW. Secondary outcomes were the identification of factors that would increase the likelihood of success, the impact of EW on discharge timing, and the incidence of adverse outcomes (lower growth velocity, return to an incubator, hospital readmission during the first week after discharge) in early weaned infants. As this was a feasibility study no formal sample size calculation could be applied, so we decided to study at least 100 infants to achieve a representative population of preterm infants. Newborns in the study period were then compared with an historical control group with similar characteristics, managed according to our weaning guidelines in 2007–2008.

### Procedures

All infants were nursed in incubators with servo-control of temperature and 60% relative humidity, from birth to transfer to open crib. Parenteral nutrition was used for infants with 1) BW ≤ 1250 2) BW ≤ 1500 g and RDS 3) gestational age (GA) <34 and small for GA. Enteral feeding with breast milk was initiated as soon as possible. Systemic blood pressure, heart rate, respiratory rate, arterial oxygen saturation (measured through pulse oximetry), and urinary output were monitored daily and recorded until the third day in the open crib. Newborns <32 weeks of gestation received caffeine until 48 hours before expected weaning from the incubator or after 1 week without documented apnea. After transition to an open crib, infants were assisted with 24°C environmental temperature and 40% relative humidity and were dressed in a woolen hat, booties, 2 vests, and a cotton wrap. The axillary temperature was measured hourly until the recording of 2 consecutive readings of ≥36.5°C, which is the normal axillary temperature in an open crib according to the American Academy of Pediatrics/American College of Obstetricians and Gynecologists perinatal guidelines [6]. If the temperature was <36.5°C, then an additional wrap was added to the infant and the temperature was checked after 2 hours. If the temperature remained at <36.5°C, then the infant was placed under a radiant warmer and the temperature was measured again after 3 hours. If the axillary temperature was still <36.5°C, then the infant was transferred back to an incubator. For infants who remained in an open crib, the axillary temperature was measured every 3 hours at feeding time, up to 72 hours after transfer. During this period, if the axillary temperature was <36.5°C in 2 consecutive readings, then the infant was transferred back to an incubator. All studied infants were weighed daily, naked, before the 9:00 AM feeding. Growth velocity was calculated according to the exponential model proposed by Patel [7]. Discharge criteria were full feeding competency (breast or bottle sucking), normal weight gain in an open crib, axillary temperature of ≥36.5°C after 72 hours, and no apneas after 72 hours without caffeine. We performed a short-term follow-up assessment after discharge home, recording data in a specific database. Four days after discharge, the following information was collected during a telephone call: body weight, general wellness of the infant, and whether the infant had required health care resources such as hospital readmissions, physician visits, or emergency department visits. The same data were collected 7 days after discharge, when the infants were seen in our pediatric ambulatory care facility.

### Statistical analysis

Continuous data were expressed as mean and standard deviation (SD) if they were normally distributed or as median and interquartile range if the normal distribution could not be accepted; categorical variables were expressed as number and percentage. We used Student’s t test and Wilcoxon rank sum test for continuous variables. The chi-squared test and the fisher’s exact test were performed for categorical variables. Logistic regression was used to identify infant baseline characteristics that would increase the likelihood of EW to a crib at lower weights. Univariate and multivariate linear regression were performed to assess the correlation of EW, GA and BW with LOS. The variance inflation factor (VIF) was calculated to evaluate the multicollinearity. P values <0.05 were considered to be statistically significant. Statistical analyses were performed with Microsoft Excel 2003 (Microsoft, Redmond, WA) and SPSS for Windows17.0 (SPSS, Chicago, IL).

## Results

During the study period 115 newborn infants with BW < 1600 g were admitted to our NICU. Ten of them died before reaching 1600 g, and 4 were excluded because of major congenital abnormalities, leaving 101 infants with a mean GA of 29.7 ± 2.6 weeks (range 23–35) and a mean BW of 1135 ± 291 grams (range 450–1590). The prevalence of male gender was 47.5% and that of small for GA infants was 23.8%. EW was successfully reached in 80 newborns, while 21 infants were transferred from incubator to an open crib at weight ≥ 1700. Table 1 compares baseline characteristics of EW versus DW. GA and BW were significantly lower in the DW group, without differences in the distribution of male and small for gestational age. The incidence of major morbidity was significantly higher in the DW group. A comparison of relevant data recorded from incubator weaning to discharge home is shown in Table 2. Weight at transfer from incubator to open crib was significantly lower in the EW. Time spent in open crib was shorter in the EW. EW infants had significantly shorter LOS, lower postmenstrual age and weight at discharge. Growth velocity in the EW was higher than in the DW. One infant in the EW required a radiant warmer, while no infants required to be transferred back to the incubator. Table 3 shows relevant data detected during the follow-up week. Growth velocity in the first 4 days and in the entire week after discharge remained higher in the EW group, so weights at 4 and 7 days after discharge were still significantly higher in these infants. Two EW infants were readmitted to the hospital for feeding intolerance problems, while one DW infant required an emergency department visit because of an upper respiratory tract infection.

**Table 1:** Baseline characteristics

	**EW**	**DW**	**p**
**n (%)**	80 (79.2%)	21 (20.8%)	
**GA, mean ± SD (range), wk**	30.3 ± 2.5	27.5 ± 2.1	< 0.0001
	(24 – 35)	(23 – 31)	
**BW, mean ± SD (range), g**	1198 ± 270	894 ± 251	< 0.0001
	(450 – 1590)	(475 – 1520)	
**Male, n (%)**	39 (48.7%)	9 (42.9%)	0.81
**SGA, n (%)**	19 (23.7%)	5 (23.8%)	1
**Necrotizing enterocolitis, n (%)**	0	3 (14.3%)	< 0.0001
**Sepsis, n (%)**	5 (6.3%)	11 (52.4%)	< 0.0001
**Intraventricular hemorrhage, n (%)**	4 (5%)	4 (19.0%)	< 0.05
**Chronic lung disease, n (%)**	0	7 (33.3%)	< 0.0001

EW early weaning; DW delayed weaning; GA gestational age; BW birth weight; SGA small for gestational age.

**Table 2:** Relevant data from incubator weaning to discharge home

	**EW**	**DW**	**p**
**Weight at transition to open crib, mean ± SD (range), g**	1621 ± 29	2047 ± 282	< 0.0001
	(1520 – 1690)	(1700 – 2670)	
**Time spent in open crib, mean ± SD (range), d**	8.6 ± 4.9	16.7 ± 19.2	0.0014
	(2 – 25)	(2 – 76)	
**LOS, median (interquartile range), d**	35	78	< 0.0001
	(26.5 - 50)	(64.2 – 121.2)	
**Postmenstrual age at discharge, mean ± SD (range), wk**	35.9 ± 1.9	40.2 ± 4.2	< 0.0001
	(33 – 44.5)	(34.3 – 50)	
**Weight at discharge, mean ± SD (range), g**	1881 ± 169	2565 ± 541	< 0.0001
	(1565 - 2515)	(1850 - 3620)	
**GV mean ± SD (range), g/kg per d**	17.5 ± 4.8	11.1 ± 7.3	< 0.0001
	(7 – 32.5)	(-8.8 – 24.2)	

EW early weaning; DW delayed weaning; LOS length of stay; GV growth velocity.

**Table 3:** Relevant data in the follow-up week

	**EW**	**DW**	**p**
**Weight after 4 d, mean ± SD (range), g**	1974 ± 188	2321 ± 335	< 0.0001
	(1590 – 2580)	(1920 – 2950)	
**GV after 4 d, mean ± SD (range), g/kg per d**	12.3 ± 9.9	4.9 ± 6.0	0.0323
	(-12-45)	(-5-15)	
**Weight after 7 d, mean ± SD (range), g**	2080 ± 228	2406 ± 290	0.0002
	(1620 – 2900)	(1960– 2920)	
**GV after 7 d, mean ± SD (range), g/kg per d**	14.5 ± 8.4	8.2 ± 7.8	0.0351
	(0-55)	(-5-22)	
**Hospital readmission, n (%)**	2 (2.5)	1 (7.7)	0.50

EW early weaning; DW delayed weaning; GV growth velocity.

Reasons for delaying transfer from incubator to open crib were persisting oxygen supplementation in 15 infants (71.4%), abdominal diseases in 5 infants (23.8%), and sepsis in 1 (4.8%).

**Figure 1:**
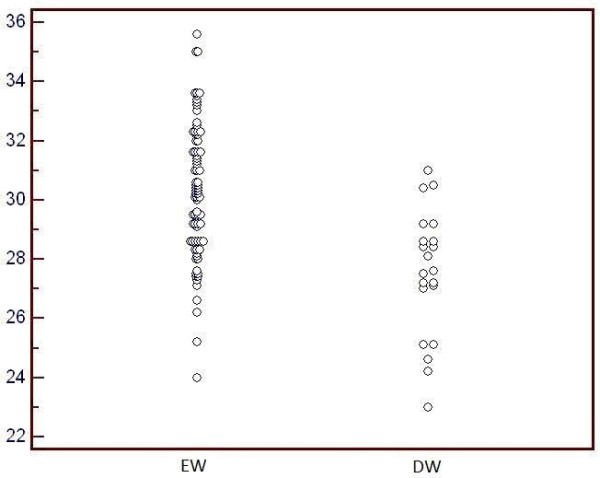
Distribution of gestational age in the early weaning (EW) and the delayed weaning group (DW).

The distribution of GA in the EW and DW group is reported in the dot diagram of Figure 1. The logistic regression evaluating how GA affected the likelihood of successful transfer into a crib at lower weights gave an OR of 1.7282; 95% CI: 1.3071 - 2.2850.

Regression analyses were used to evaluate the relationship of LOS with the following independent variables: GA, BW, EW. Independent variables with a p value < 0.25 in the univariate model were entered into a multiple regression model. All the variables analyzed were significantly associated with LOS in the multiple regression analysis: GA (-2.6 d/wk increase in GA, p = 0.0003, VIF = 1.880), BW (-5.4 d/100 grams increase in BW, p < 0.0001; VIF = 1.865), EW (-25.8 d p < 0.0001; VIF = 1.281). The Coefficient of determination (R^2^) was 0.81.

We finally compared the entire population (EW plus DW) of the studied newborns (study group: SG) with a control group (CG) of infants admitted to our NICU in the period 2007–2008, when infants were transferred to open crib at ≥ 1800 g. (Table 4). Baseline characteristics were similar, but LOS (40 vs 46.5 days; p = 0.0163) and postmenstrual age at discharge (36.8 vs 37.8 wks; p = 0.0131) were significantly lower in the SG.

**Table 4:** Comparison with historical controls

	**SG**	**CG**	**p**
**n**	101	120	
**GA, mean ± SD (range), w**	29.7 ± 2.6	29.4 ± 2.5	0.3767
	(23 – 35)	(23 – 36)	
**BW, mean ± SD (range), g**	1135 ± 292	1138 ± 284	0.9365
	(450 – 1590)	(500 – 1590)	
**CRIB score (mean ± SD)**	2.3 ± 0.4	2.1 ± 0.5	0.847
**Male, n (%)**	48 (47.5%)	63 (52.5%)	0.500
**SGA, n (%)**	24 (23.8%)	35 (29.1%)	0.446
**LOS, median (interquartile range), d**	40	46.5	0.0163
	(29.5-61.7)	(37-65.5)	
**Postmenstrual age at discharge, mean ± SD (range), wk**	36.8 ± 3.0	37.8 ± 2.7	0.0131
	(33.0-50.0)	(35.0-50.0)	

SG study group; CG control group; GA gestational age; BW birth weight; SGA small for gestational age; LOS length of stay.

## Discussion

This study demonstrated that early weaning was feasible and safe in a population of preterm infants. In fact, 79.2% of the premature newborns admitted to our NICU were successfully transferred to open crib between 1.600 and 1699 g without significant adverse effects on temperature stability or weight gain, and none of them required to be transferred back to the incubator. Transfer from incubator was delayed mainly because of the need of respiratory support. A low GA implies a low probability to be successfully transferred at 1600 g. GA in the DW was almost 3 weeks lower than in the EW, and only 4:9 infants with GA < 27 weeks were successfully weaned at 1600 g. Logistic regression analysis demonstrated that every additional week in GA over 27 increased the likelihood of success by 1.7 fold.

Reduction of LOS is an important goal to be achieved for preterm infants because early discharge improves parental bonding, prevents NICU overcrowding, reduces exposure to nosocomial infections, and has important economic implications [8-10]. The EPIPAGE study demonstrated that preterm infants discharged with lower weight and postmenstrual age did not have higher rates of re-hospitalization, and that the number of re-admissions was not correlated with the initial LOS [11]. It seems conceivable that LOS can be influenced by the age in which the newborn leaves the incubator. Picone et al. recently reported a retrospective study on 234 newborns < 32 weeks of GA showing that an early transfer from incubator at a mean weight of 1737 g promoted an early discharge from hospital at a mean weight of 1900, without adverse effect on post-discharge weight gain and re-hospitalization rate [12]. The randomized clinical trial, conducted in the neonatal sub-intensive ward of our university hospital, demonstrated that weaning moderately preterm infants at a body weight of 1600 g versus 1800 g reduced the median LOS of 9.5 days [4], while New et al. failed to find shorter LOS in infants transferred at lower weights [3,5]. The multiple linear regression analysis showed that GA, BW and EW itself were significantly associated with LOS. In fact, every additional week in GA reduced LOS by 2.6 days, each 100 g increase in BW reduced LOS by 5.4 days, and the EW reduced LOS by 25.8 days. These three variables explained 81% of the variation in LOS. The impact of early transfer on LOS was further confirmed by the comparison between SG and CG. Even though general characteristics, and severity as indicated by the CRIB score were similar, the median LOS was 6.5 days shorter in the SG.

Apart from the studies of New et al. and Zecca et al. [4,5] there are no randomized controlled trials concerning weaning protocols from incubator. Sutter and colleagues [13] showed that preterm infants with BW > 1000 g could be safely transferred to open cribs at 1700 grams. Medoff-Cooper [14] studied 270 infants with a mean BW of 1188 g and a mean GA of 29.3, who were moved to an open crib at a mean weight of 1598 g. West et al. [15] reported no differences in weight gain rate, transfer failure rates, temperature controls ability between the four cohorts of infants who were transferred to open crib at 1800 g, 1700 g, 1600 g, 1500 g. Schneiderman et al. [15] conducted an observational study of 2908 preterm infants in 579 hospital throughout United States, and found that delays in timely incubator weaning to an open crib was associated with delayed time to achieve full-volume oral feeds, decreased growth velocity and prolonged LOS.

Our study has some limitation: it is a non-randomized investigation, and the comparison of outcomes was performed with historical controls. The strengths of our study are the characteristics of the studied population, well representing the characteristics of premature newborns, and the clinical follow-up after discharge that was performed by our ambulatory care facility. It is also noteworthy that it is a prospective feasibility study in which all babies admitted to our NICU were studied, thus avoiding sampling bias. In conclusion, our study shows that preterm infants admitted to NICU can be weaned from an incubator to an open crib at weight as low as 1600 grams, reducing LOS without significant adverse effect.

## Abbreviations

BW: Birth weight; CG: Control group; DW: Delayed weaning; EW: Early weaning; GA: Gestational age; LOS: Length of stay; SG: Study group; VIF: Variance inflation factor.

## Competing interests

The authors declare that they have no competing interests.

## Authors’ contributions

EZ designed the study and has made substantial contributions in drafting manuscript. MC, FP and PP performed data acquisition and contributed to interpretation of data. CR revised the manuscript critically. GB performed statistical analysis and has made substantial contributions in drafting manuscript. All authors read and approved the final manuscript.
